# Environmental control on the distribution of metabolic strategies of benthic microbial mats in Lake Fryxell, Antarctica

**DOI:** 10.1371/journal.pone.0231053

**Published:** 2020-04-13

**Authors:** Megan L. Dillon, Ian Hawes, Anne D. Jungblut, Tyler J. Mackey, Jonathan A. Eisen, Peter T. Doran, Dawn Y. Sumner

**Affiliations:** 1 Ecology Department, Lawrence Berkeley National Laboratory, Berkeley, CA, United States of America; 2 Department of Earth and Planetary Sciences, University of California, Davis, Davis, California, United States of America; 3 Coastal Marine Field Station, University of Waikato, Hamilton, Waikato, New Zealand; 4 Life Sciences Department, Natural History Museum, London, England, United Kingdom; 5 Department of Earth, Atmospheric, and Planetary Sciences, Massachusetts Institute of Technology, Cambridge, Massachusetts, United States of America; 6 Department of Evolution and Ecology, University of California, Davis, Davis, California, United States of America; 7 Geology and Geophysics Department, Louisiana State University, Baton Rouge, Louisiana, United States of America; University of Siena, ITALY

## Abstract

Ecological theories posit that heterogeneity in environmental conditions greatly affects community structure and function. However, the degree to which ecological theory developed using plant- and animal-dominated systems applies to microbiomes is unclear. Investigating the metabolic strategies found in microbiomes are particularly informative for testing the universality of ecological theories because microorganisms have far wider metabolic capacity than plants and animals. We used metagenomic analyses to explore the relationships between the energy and physicochemical gradients in Lake Fryxell and the metabolic capacity of its benthic microbiome. Statistical analysis of the relative abundance of metabolic marker genes and gene family diversity shows that oxygenic photosynthesis, carbon fixation, and flavin-based electron bifurcation differentiate mats growing in different environmental conditions. The pattern of gene family diversity points to the likely importance of temporal environmental heterogeneity in addition to resource gradients. Overall, we found that the environmental heterogeneity of photosynthetically active radiation (PAR) and oxygen concentration ([O_2_]) in Lake Fryxell provide the framework by which metabolic diversity and composition of the community is structured, in accordance with its phylogenetic structure. The organization of the resulting microbial ecosystems are consistent with the maximum power principle and the species sorting model.

## Introduction

The microbial components of ecological communities (the microbiome) provide a large proportion of the genetic novelty and perform a large proportion of the functions of an ecosystem (for example, [1–3]). However, many of the methods that are used to explore microbiomes were developed by investigating plant- and animal-dominated ecosystems [4]. The composition, assembly, and function of microbiomes are considerably more complex than macroscopic processes because there are more individuals, more populations, and thus more possible interactions. Further, the phylogenetic relationships and metabolic capacity of microorganisms are often fundamentally different from plants and animals (*e*.*g*., horizontal gene transfer and mixotrophy). If the ecological theories developed from well-studied macroscopic ecosystems are universal, they should apply to microbial ecosystems. Therefore, we can gain insights into the general applicability of ecological theories by studying microbiomes [5–7].

In all ecosystems, heterogeneity in environmental conditions can greatly affect community membership. The extent to which this holds depends on the extent to which niche selection, drift, speciation or mutation, and dispersal affect taxonomic and functional richness, evenness, and composition [8]. The explicit effects of these processes have been developed into a metacommunity framework, applicable across spatial scales, and give rise to alternative models (species sorting, neutral theory, patch dynamics, and mass effects), depending on the degree to which each is relevant for a given ecosystem [9,10].

In the *species sorting* model [10], fitness advantages of existing community members limit the survival and growth of immigrants to only those that are most competitive. Therefore, environments with greater habitat heterogeneity have more diverse fitness landscapes and are thus inhabited by a more diverse community than homogenous habitats. Communities that conform to the species sorting model are ones in which species are distributed according to local environmental conditions and community heterogeneity matches environmental heterogeneity. Drift, speciation or mutation, and dispersal effects are damped out by niche selection. Thus, under the species sorting model, we expect to observe community composition changing in response to habitat features, and total habitat heterogeneity in a landscape directly influences community diversity. The species sorting model is the most widely cited as important in shaping microbial community dynamics, especially in aquatic ecosystems [11–13].

In contrast, the *neutral theory* model assumes that all organisms in a community are equally suited for their habitat. Therefore drift, speciation or mutation, and dispersal processes dominate over niche selection and community variations do not reflect variations in environment. Thus, neutral theory models produce a wide range of communities that are randomly distributed across heterogeneous habitats. Neutral dynamics have successfully described microbial community assembly in host-associated microbiome studies [14–16].

The *patch dynamics* model assumes homogeneous local environments where species coexist due to stochastic extinction and advantageous dispersal. In this model, community composition depends on early colonizers, which induce priority effects [17]. Differences in niche and fitness may exist among community members, but drift and dispersal dominate, leading to a community that does not depend on environmental heterogeneities. Under the patch dynamics model, populations are randomly distributed across relatively homogenous habitats, with greater diversity than expected from the homogenous landscape. Patch dynamics models are consistent with some studies of the human microbiome, which show significant diversity across individual patients or body sites that are interpreted as due to colonization history [18].

Finally, the *mass effects* model applies when dispersal overwhelms and masks selection, drift, and speciation or mutation, creating uniform communities composed of the same dominant organisms irrespective of environment. The mass effects model produces ecosystems in which community composition varies across different habitats and geography according to dispersal from parent communities. For example, the microbial composition of arctic streams are similar to the soils from which they originate near headwaters due to mass effects, but change as geographic distance and environmental heterogeneity increase [19].

The metacommunity framework explicitly describes community membership, but necessarily applies to functional aspects of communities as well. The means by which selection, drift, mutation, and dispersal affect community membership are through individuals' traits. The *species sorting*, *neutral theory*, *patch dynamics*, and *mass effects* models all hinge on the relative fitness of community members, which is determined by how well their phenotypes (functions) allow them to survive and reproduce in a given habitat or under specific environmental conditions.

These metacommunity models can be used to understand ecological processes in microbial communities that lack macroscopic organisms. Specifically, microbial ecosystems in ice-covered lakes in the McMurdo Dry Valleys (MDVs), Antarctica, serve as natural laboratories to test the extent to which these models can explain community variations as a function of environmental gradients in photosynthetically active radiation (PAR) and oxygen concentration ([O_2_]). The MDV lake environments are stable on decade-long timescales [20,21], containing well characterized PAR and slowly changing [O_2_] gradients, which lead to predictable habitat heterogeneity [22,23]. PAR and [O_2_] gradients are particularly prominent in Lake Fryxell, a perennially ice-covered, density-stratified lake in the Taylor Valley, Antarctica.

Our prior investigations into the relationships between the phylogenetic structure and taxonomic composition of Lake Fryxell’s benthic microbial mats and local environmental conditions demonstrated that PAR and [O_2_] affect local community membership differently at mm- and m-scales [23]. At the mm-scale, phototrophs dominate top mat layers where they maximize conversion of PAR into chemical energy and suppress α-diversity due to their high population [23]. The phylogenetic diversity of the underlying non-phototrophic layers increases with depth into the mat, consistent with the maximum power principle, which predicts that communities are structured to optimize energy consumption over time [23,24]. In mat layers where [O_2_] was saturating, PAR structured the community. At the m-scale however, [O_2_] positively correlated with diversity and affected the distribution of dominant populations across the three habitats. This suggests that meter-scale diversity is structured by PAR, as predicted by species-energy theory, which posits that areas with greater net primary productivity have more diverse habitats [4,25].

Because both the maximum power principle and species-energy theory require niche selection, prior results suggest that the species sorting model may be most appropriate for describing the benthic mat structure in Lake Fryxell across large- and small-scale PAR and [O_2_] gradients. Neutral theory models are not appropriate because the communities systematically vary along environmental gradients. Similarly, the stratification of lake water means that the transport of organisms within Lake Fryxell is likely too low for populations to be controlled by mass effects. Finally, since the landscape features (PAR, [O_2_]) are heterogeneous, the patch dynamics model does not apply [23].

Because species sorting was found to be an appropriate model for the phylogenetic diversity and taxonomic composition of Lake Fryxell’s benthic microbial mats, we tested whether patterns of metabolic capacity reflect the environmental conditions in Lake Fryxell across lake depth and through mat layers, also consistent with the species sorting model. Recent work has found that different ecological processes may influence phylogenetic and metabolic composition and diversity in microbial communities [26]. Indeed, due to the modular structure of cellular biochemistry [27], it may be the case that metabolic structure is more directly affected by environmental conditions than phylogenetic structure, which is additionally influenced by species-species interactions [28]. Application of the species sorting model to metabolic capacities would mean that the local distributions of PAR and [O_2_] dictate the local metabolic capacity of the mats, similar to the distribution of species.

## Materials and methods

### Site description

Lake Fryxell (77°36´S 162°6´ E) is a physically stratified, low-nutrient habitat in the McMurdo Dry Valleys (MDV), Antarctica. It is 5 km x 1.5 km in extent and the maximum depth is approximately 20 m [20]. Water is supplied to Lake Fryxell by 13 glacial melt-water streams primarily sourced from the Canada and Commonwealth glaciers [29]. Water balance is achieved by evaporation and ablation from the surface; there are no out-flowing streams [30].

**Fig 1:**
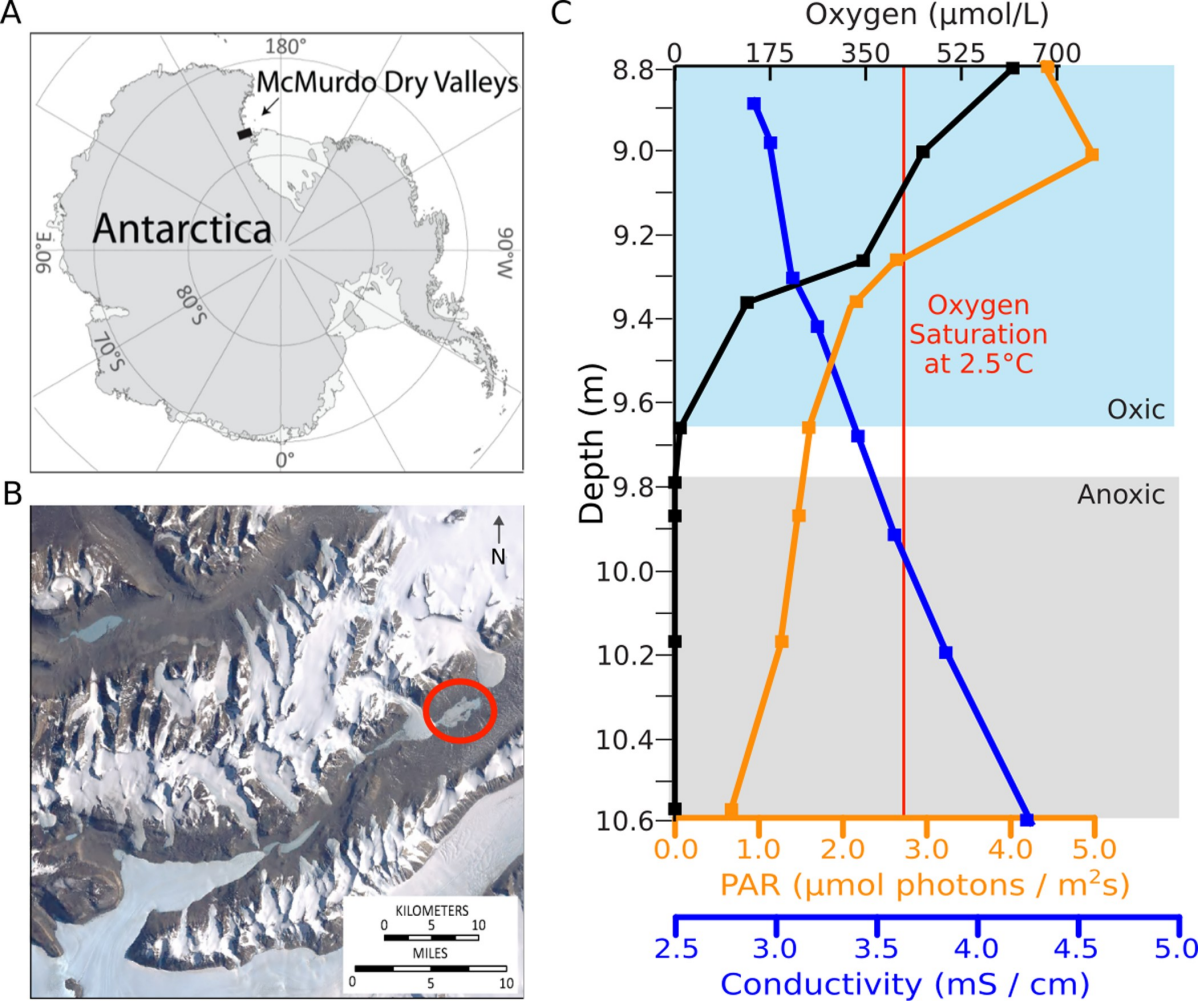
Sampling site and environmental conditions. A) The McMurdo Dry Valleys are in Southern Victorialand, East Antarctica [35]. B) Location of Lake Fryxell in Taylor Valley (circled in red) [36]. C) Oxygen concentration, conductivity, PAR, and oxygen saturation at 0°C along a benthic mat transect in Lake Fryxell in November 2012 [22]. The linear increase in conductivity indicates stably density-stratified waters, and the oxygen saturation line shows areas of the lake that are oxygen-supersaturated.

Environmental conditions in Lake Fryxell are strongly affected by a 4–5 m thick perennial ice cover [31]. During the summer, the ice cover transmits approximately 1% of incident irradiance [22], which provides the lake’s primary energy influx. Light reaching the benthic surface of Lake Fryxell declines with increasing depth in the water column but is adequate to support photosynthesis in surface layers under anoxic water to depths of 10.4 m during the summer months (Fig 1). The ice cover inhibits wind mixing and gas equilibration between lake water and the atmosphere. The lack of mixing produces stable density stratification, as demonstrated by conductivity profiles [21,22] (Fig 1). The stratification limits the transport of nutrients and redox pairs to diffusion and creates stable redox and nutrient gradients in the water column [22,32]. Temperature varies from 2.4 to 2.7°C and pH varies from 7.50 to 7.52 along a lake-bottom transect through the oxycline [22]. As lake water freezes during winter, oxygen and other gases are excluded from the underside of the ice cover, building to gas supersaturation in shallow waters. Oxygen concentration declines with depth, and oxygen is absent from the water column below approximately 9.8 m. The oxygen limit is therefore partially determined by the ice cover.

Lake Fryxell’s robust planktonic microbial community thrives near the oxic-anoxic transition (9–10 m), coincident with the deep chlorophyll maximum and the nutricline [33]. Centimeter-to-decimeter-scale thick microbial mats exhibiting a variety of pigments and morphologies grow on the benthic surface of the lake to depths of at least 10.5 m [22], affecting the seasonal redox conditions near the oxycline via oxygenic photosynthesis and respiration. In late spring, a seasonal oxygen oasis forms at approximately 9.8 m and [O_2_] varies significantly through the microbial mats by lake depth, according to microelectrode profiles: 650–825 μmol O_2_ / L to at 9.0 m, and 0–50 μmol O_2_ / L at 9.8 m [34].

### Sampling

The benthic microbial mats in Lake Fryxell were sampled in November 2012 as permitted by the New Zealand Minister of Foreign Affairs, described by Jungblut *et al*. (2016). Sampling was performed at 9.0, 9.3, and 9.8 m depths along a transect that was installed in 2006 [37]. At 9.0 m, top layers were exposed to PAR, and middle and bottom layers were not exposed to PAR; [O_2_] was saturated in all layers [22,34]. At 9.3 m, all layers were exposed to PAR due to mat topography; top and middle layers were exposed to oxygenated water, but the bottom layers were anoxic [22]. At 9.8 m, film and top layers were exposed to PAR; film and top samples were seasonally exposed to O_2_ [22,34]. All sampling and dissection were performed using sterile technique. Divers retrieved samples from the bottom of the lake by cutting samples out of *in situ* mats using a spatula and lifting them into plastic boxes underwater. Upon delivery to the surface, multiple samples from each depth were dissected according to layer pigmentation and morphology. The samples were preserved in the field immediately after sampling using an Xpedition Soil/Fecal DNA MiniPrep kit (Zymo Research, Irvine, CA), stored on ice for the remainder of the field season, and shipped frozen to University of California, Davis where they were stored at -80°C until DNA was extracted [22].

### Metagenomic sequencing

DNA was extracted using an Xpedition Soil/Fecal DNA MiniPrep kit (Zymo Research, Irvine, CA) as per manufacturer instructions from biological and technical replicates of 10 sample types (S1 Table; [23]). Metagenomic sequencing was performed at the University of California, Davis Genome Center DNA Technologies Core (http://dnatech.genomecenter.ucdavis.edu/) using the Illumina HiSeq 2500, PE 250 platform. Library preparation was performed using Illumina’s Nextera DNA Kit (Oligonucleotide sequences © 2007–2013 Illumina, Inc.). Reads were quality filtered to Q20, and forward and reverse reads were joined using PEAR v0.9.6 [38]. Downstream analyses included only biological replicates with greater than 10,000 reads.

### Bioinformatics

Humann2 [39] was used to characterize metabolic genes from all domains, using the ChocoPhlan and UniRef databases. The comprehensive UniRef50 clusters [40] were used within Humann2 to identify proteins. Gene families discovered using Humann2 were normalized using copies per million (CPM), which allows a direct comparison across samples [39].

The distribution of specific metabolic pathways was evaluated by comparing the proportion of metabolic marker genes mapping to each community. Microbial metabolism drives the biogeochemical cycles of all major elements on Earth, including the oxygen, carbon, nitrogen, and sulfur cycles [41–48]. We chose genes within these pathways as representative of major metabolic processes (nitrogen fixation, the Calvin Cycle, oxygenic photosynthesis, etc). Genes marking metabolisms of interest (Table 1) were chosen for their lack of pathway ambiguity, phylogenetic breadth, and importance in major element cycles. Gene families were regrouped and assigned to their Kyoto Encyclopedia of Genes and Genomes (KEGG) orthology (KO) [49].

**Table 1:** Metabolic marker genes.

General Process	Specific Process	Gene Product	Gene	KEGG ID	KEGG Description
Photosynthesis	Anoxygenic Photosynthesis	PSII	*pufL*	K08928	Photosynthetic Reaction Center L Subunit
		PSI	*pscA*	K08940	Photosystem P840 Reaction Center Large Subunit
	Oxygenic Photosynthesis	PSII—P680	*psbA*	K02703	Photosystem II P680 Reaction Center D1 Protein [EC:1.10.3.9]
Carbon Cycling	Carbon Fixation	Ribulose Bisphosphate Carboxylase Complex	*rbcL*	K01601	Ribulose-Bisphosphate Carboxylase Large Chain [EC:4.1.1.39]
	Methanotrophy	Methanol Dehydrogenase	*mdh2*	K14029	Methanol Dehydrogenase (Cytochrome C) Subunit 2
	Methanogenesis	Heterodisulphide Reductase	*hdrD*	K08264	Heterodisulfide Reductase Subunit D
		Heterodisulphide Reductase	hdrB*	K03389	Heterodisulfide Reductase Subunit B [EC:1.8.98.1]
	Aerobic Respiration	Cytochrome C Oxidase	*ccoNO*	K15862	Cytochrome C Oxidase Cbb3-Type Subunit I/II
Nutrient Assimilation	Polysaccharide Hydrolysis	Amylase	*amyA*	K01176	Alpha-Amylase
	Nitrogen Fixation	Nitrogenase	*nifH*	K02588	Nitrogenase Iron Protein [EC:1.18.6.1]
	Assimilatory Sulfate Reduction	Sulfite Reductase	*cysI*	K00381	Sulfite Reductase (NADPH) Hemoprotein Beta-Component
	Assimilatory Nitrate Reduction	Nitrite Reductase	*nasA*	K00372	Assimilatory Nitrate Reductase Catalytic Subunit
Anaerobic Respiration	Denitrification	N2O Reductase	*nosZ*	K00376	Nitrous-Oxide Reductase [EC:1.7.2.4]
	Respiratory Nitrate Reduction	Nitrite Reductase	*nrfA*	K03385	Nitrite Reductase (Cytochrome C-552)
	Nitrification	Hydroxylamine Dehydrogenase	*hao*	K10535	Hydroxylamine Dehydrogenase
	Respiratory Sulfate Reduction	Adenylylsulfate Reductase	*aprB*	K00395	Adenylylsulfate Reductase, Subunit B
	Dimethyl Sulfide Generation	Sulfane Dehydrogenase	*soxC*	K17225	Sulfane Dehydrogenase Subunit
Phosphorus Limitation	Substitution of Nitrogen for Phosphorus in Membrane Lipids	Glycosyltransferase	*btaA*	K13622	S-Adenosylmethionine-Diacylglycerol 3-Amino-3-Carboxypropyl Transferase

Genes of interest were subset from all gene families called by Humann2.

*The gene *hdrB* also indicates flavin-based electron bifurcation (see text).

When calculating CPM, unmapped and ungrouped reads were carried forward. Unmapped reads are those which did not align during either nucleotide or translated searches. Ungrouped reads are those that did not match any features in KEGG [39]. CPM for reads that both mapped and grouped was then normalized to percent grouped for downstream analyses.

### Statistical analyses

Alpha diversity was calculated using Simpson’s index of diversity directly on gene families, as called by Humann2. Significant differences in metabolic marker genes, and gene family alpha diversity between samples were determined using Permutational Multiple Analysis of Variance (PERMANOVA) in R v3.3.2 [50–52] using R package vegan v2.5–5 [23,53]. Samples determined to differ significantly in alpha diversity, as per PERMANOVA implemented via the adonis function, were then subjected to Tukey’s Honest Significant Difference (Tukey’s HSD) test [54] in R v3.3.2 [52] to establish which genes differed between depths and between layers at each depth.

## Results

### Bioinformatics

Metagenomic sequencing yielded approximately 5 x 10^9^ bp per sample type (S2 Table). On average, approximately 34% of the metagenomic reads mapped to the RefSeq50 database. Of the reads that mapped, approximately 74% were grouped in KEGG as KOs (Table 2). Approximately 8% of total reads mapped to the RefSeq50 database and grouped as KOs.

**Table 2:** Mapping and grouping of reads.

Sample	Unmapped (% CPM)	Ungrouped (% CPM)	Mapped & Grouped (% CPM)
9.0 m			
Top	76.68	18.74	4.58
Middle	70.27	23.01	6.72
Bottom	69.13	23.92	6.95
9.3 m			
Top	73.47	21.08	5.45
Middle	66.91	25.65	7.44
Bottom	65.36	26.37	8.28
9.8 m			
Film	35.03	45.22	19.75
Top	53.11	33.91	12.97
Middle	60.84	29.25	9.91
Bottom	66.91	25.95	7.14

Average percent counts per million of unmapped (to the RefSeq50 database), ungrouped (to KEGG KO identifiers, but mapped to the RefSeq50 database), and mapped and grouped reads per sample.

### Gene family diversity

**Fig 2:**
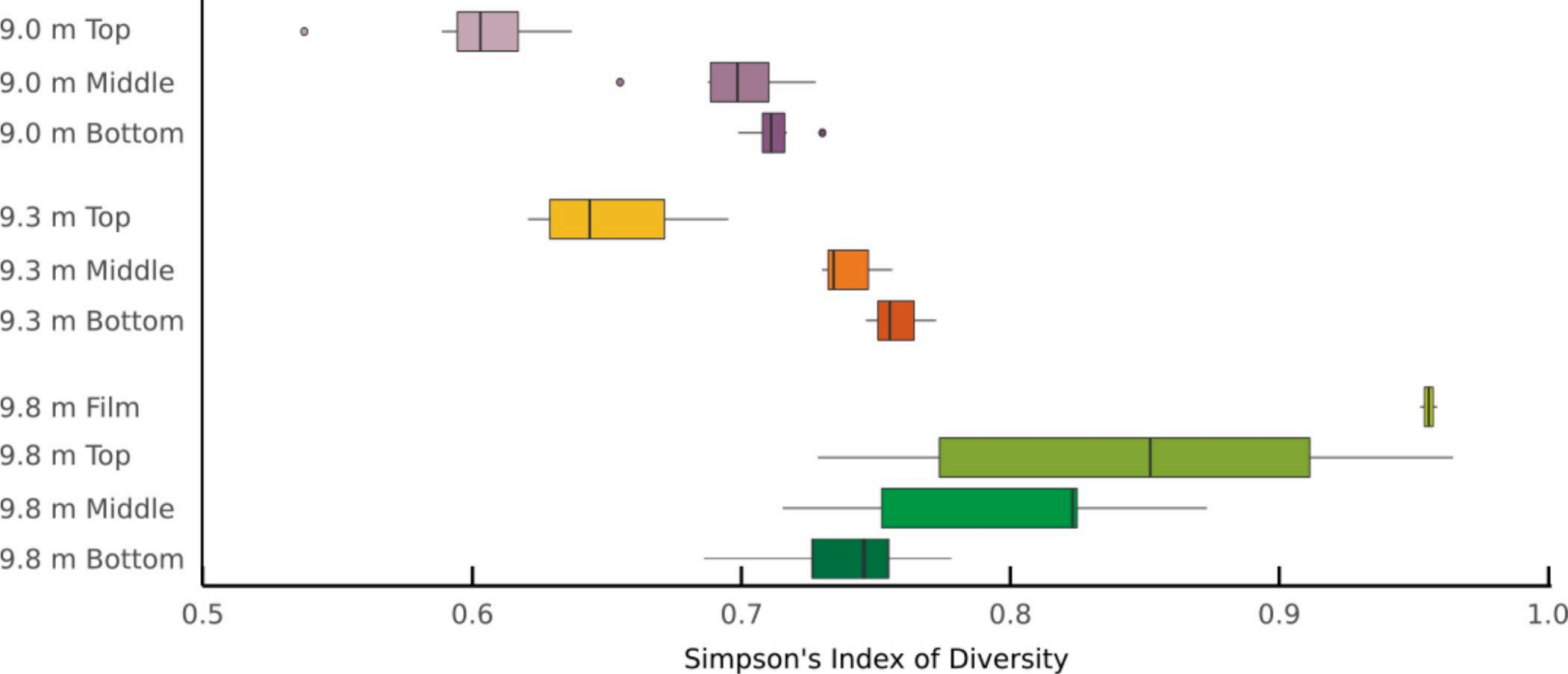
Alpha diversity. Alpha diversity (Simpson’s Index of Diversity of gene families, as called by Humann2) of samples increases through mat layers at 9.0 and 9.3 m depths but decreases at 9.8 m. Boxplots display the median and first and third quartiles, and the whiskers extend to 1.5 times the interquartile range (IQR). Points outside 1.5⨉IQR are shown individually.

Gene family diversity varied with lake depth and mat layer, from approximately 0.6 to 0.95, as measured by Simpson's Index of Diversity (Fig 2). ANOVA demonstrated several key differences in the diversity of genes present across depths and layers (Table 3). At 9.0 m, the top layer is significantly less diverse than all other samples except the top layer at 9.3 m. At 9.8 m, the film and top layers are significantly more diverse than all other samples. Gene family diversity increased with lake depth. At 9.0 and 9.3 m, alpha diversity increased from the top to bottom layers, whereas at 9.8 m, alpha diversity decreased through mat layers (Fig 2). Phylogenetic and metabolic diversity are correlated in only three samples (S3 Table).

**Table 3:** Gene family alpha diversity.

	9.0 m			9.3 m			9.8 m			
	Top	Middle	Bottom	Top	Middle	Bottom	Film	Top	Middle	Bottom
9.0 m										
Top	x									
Middle	0.0006	x								
Bottom	0.0002	NS	x							
9.3 m										
Top	NS	NS	NS	x						
Middle	<0.0001	NS	NS	0.0041	x					
Bottom	<0.0001	NS	NS	0.0003	NS	x				
9.8 m										
Film	<0.0001	<0.0001	<0.0001	<0.0001	<0.0001	<0.0001	x			
Top	<0.0001	<0.0001	<0.0001	<0.0001	0.0005	0.0070	NS	x		
Middle	<0.0001	0.0017	NS	<0.0001	NS	NS	0.0006	NS	x	
Bottom	<0.0001	NS	NS	0.0048	NS	NS	<0.0001	0.0004	NS	x

Significant differences (adjusted P-values, Tukey’s honest significant difference test) in gene family alpha diversity, calculated as Simpson’s index of diversity directly on gene families as called by Humann2, between sample types. Entries with P-values greater than 0.01 are marked "NS" and self-comparisons are marked “x”.

### Metabolic marker gene presence, absence, and relative abundance

To explore how gene family diversity correlated with environmental parameters, specific metabolic marker genes (Table 1) were chosen to represent distinct metabolic strategies. Some samples lacked one or more of the genes representing these strategies, and where metabolic genes were present, their relative abundances varied among depths and mat layers (Table 4).

**Table 4:** Average relative abundances.

Sample	Oxygenic Photosynthesis (psbA)	Anoxygenic Photosynthesis (pufL)	Carbon Fixation (rbcL)	Methanotrophy (mdh2)	Aerobic Respiration (ccoNO)	Methanogenesis (hdrB)	Nitrogen Fixation (nifH)	Polysaccharide Hydrolysis (amyA)	Assimilatory Nitrate Reduction (nasA)	Assimilatory Sulfate Reduction (cysI)	Nitrification (hao)	Dimethyl Sulfide Generation (soxC)	Respiratory Sulfate Reduction (aprB)	Denitrification (nosZ)	Respiratory Nitrate Reduction (nrfA)	Membrane Phosphorus Substitution (btaA)
9.0 m																
Top	61.43	0.00	34.57	0.00	13.36	0.00	0.00	28.00	8.57	20.71	0.00	2.57	0.00	7.29	10.71	3.14
Middle	25.88	0.75	11.50	0.00	14.56	0.00	0.00	14.75	18.25	27.38	0.00	4.75	2.38	24.50	30.13	5.13
Bottom	4.67	0.00	4.50	0.00	12.67	0.33	0.00	0.83	16.50	29.17	0.50	9.50	1.50	32.83	29.83	0.67
9.3 m																
Top	23.14	0.00	13.14	0.00	18.21	5.86	3.00	6.71	11.00	27.29	0.00	5.86	6.14	19.14	23.86	4.71
Middle	2.57	0.29	10.67	0.00	21.57	3.86	2.71	2.29	18.29	34.29	1.29	8.43	9.14	31.57	31.57	0.57
Bottom	0.14	0.00	6.71	0.00	14.50	7.57	2.71	1.00	18.29	32.86	0.86	12.14	10.71	26.57	25.57	0.00
9.8 m																
Film	753.00	0.00	232.50	2.50	10.25	245.00	0.00	0.50	13.00	12.00	1.50	2.00	8.00	12.00	17.00	110.50
Top	465.83	0.00	143.83	0.00	15.08	108.83	5.67	0.67	13.50	21.33	0.00	3.83	7.83	20.00	21.33	62.83
Middle	261.60	0.00	94.00	0.00	19.90	64.40	4.20	1.60	15.60	26.00	0.00	7.20	2.20	17.40	28.80	46.60
Bottom	13.71	0.00	16.71	0.00	26.36	12.71	0.00	0.86	23.14	39.00	0.00	8.71	27.71	25.57	36.71	2.71

Replicate averages of metabolic marker gene counts per million by sample.

**Fig 3:**
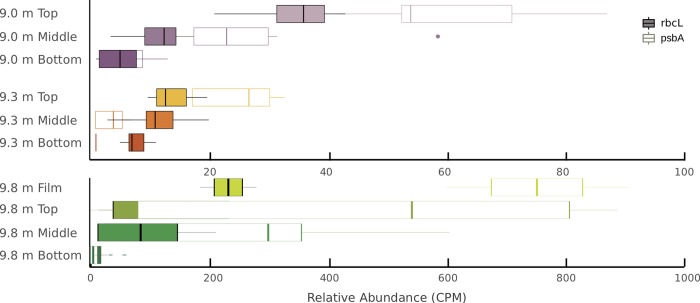
Relative abundances of oxygenic photosynthesis and carbon fixation marker genes. *psbA (*open boxes) and *rbcL(*filled boxes) genes in counts per million from sample metagenomes. Boxplots display the median and first and third quartiles, and the whiskers extend to 1.5 times the interquartile range (IQR). Points outside 1.5⨉IQR are shown individually.

The predicted capacity to perform photosynthesis and fix carbon decreases through mat layers at all depths (Fig 3). The potential for oxygenic photosynthesis (*psbA*) was present in all sample types; however, the relative abundance of this gene was greater in top, illuminated layers than in dark bottom layers at all depths (Fig 3 and Table 4). Similarly, Calvin cycle carbon fixation (*rbcL*) was consistently present in all samples and decreased in relative abundance through the layers at all depths (Table 4). The capacity for oxygenic photosynthesis and carbon fixation were strongly correlated (Fig 3 and Table 5). This correlation is unsurprising considering that both genes are often present in organisms capable of oxygenic photosynthesis [55], though *rbcL* is not found exclusively in oxygenic phototrophs. Little evidence of the capacity for anoxygenic photosynthesis (*pufL*) was found; *pufL was* only identified in middle layers at 9.0 and 9.3 m where PAR is very low (Table 4). The capacity for alternative anoxygenic photosynthesis strategies (*pscA*) were absent from all samples. Additionally, the capacity for polysaccharide hydrolysis (*amyA*) was present in all samples and had the highest relative abundance where PAR was highest (Table 4).

**Table 5:** Metabolic marker gene correlations (Pearson's correlation coefficient). Self-comparisons marked "x".

	psbA	pufL	rbcL	mdh2	ccoNO	hdrB	nifH	amyA	nasA	cysI	hao	soxC	aprB	nosZ	nrfA	btaA
psbA	x															
pufL	-0.26	x														
rbcL	0.998	-0.28	x													
mdh2	0.807	-0.149	0.802	x												
ccoNO	-0.427	-0.013	-0.401	-0.465	x											
hdrB	0.983	-0.262	0.979	0.89	-0.405	x										
nifH	0.178	-0.24	0.176	-0.305	0.185	0.077	x									
amyA	-0.279	0.291	-0.258	-0.204	-0.204	-0.344	-0.381	x								
nasA	-0.338	0.296	-0.345	-0.217	0.584	-0.257	-0.142	-0.514	x							
cysI	-0.805	0.141	-0.803	-0.684	0.755	-0.754	0.001	-0.235	0.759	x						
hao	0.338	-0.044	0.327	0.641	-0.273	0.457	-0.156	-0.389	0.101	-0.169	x					
soxC	-0.62	-0.104	-0.627	-0.484	0.406	-0.547	0.103	-0.485	0.667	0.82	0.12	x				
aprB	-0.072	-0.196	-0.065	0.019	0.674	0.012	-0.082	-0.444	0.672	0.545	0.056	0.343	x			
nosZ	-0.526	0.277	-0.557	-0.418	0.337	-0.47	0.031	-0.532	0.736	0.746	0.183	0.78	0.27	x		
nrfA	-0.463	0.308	-0.473	-0.394	0.69	-0.403	0.007	-0.54	0.893	0.814	-0.071	0.679	0.507	0.817	x	
btaA	0.995	-0.249	0.994	0.809	-0.39	0.983	0.188	-0.322	-0.3	-0.78	0.34	-0.584	-0.07	-0.505	-0.407	x

**Fig 4:**
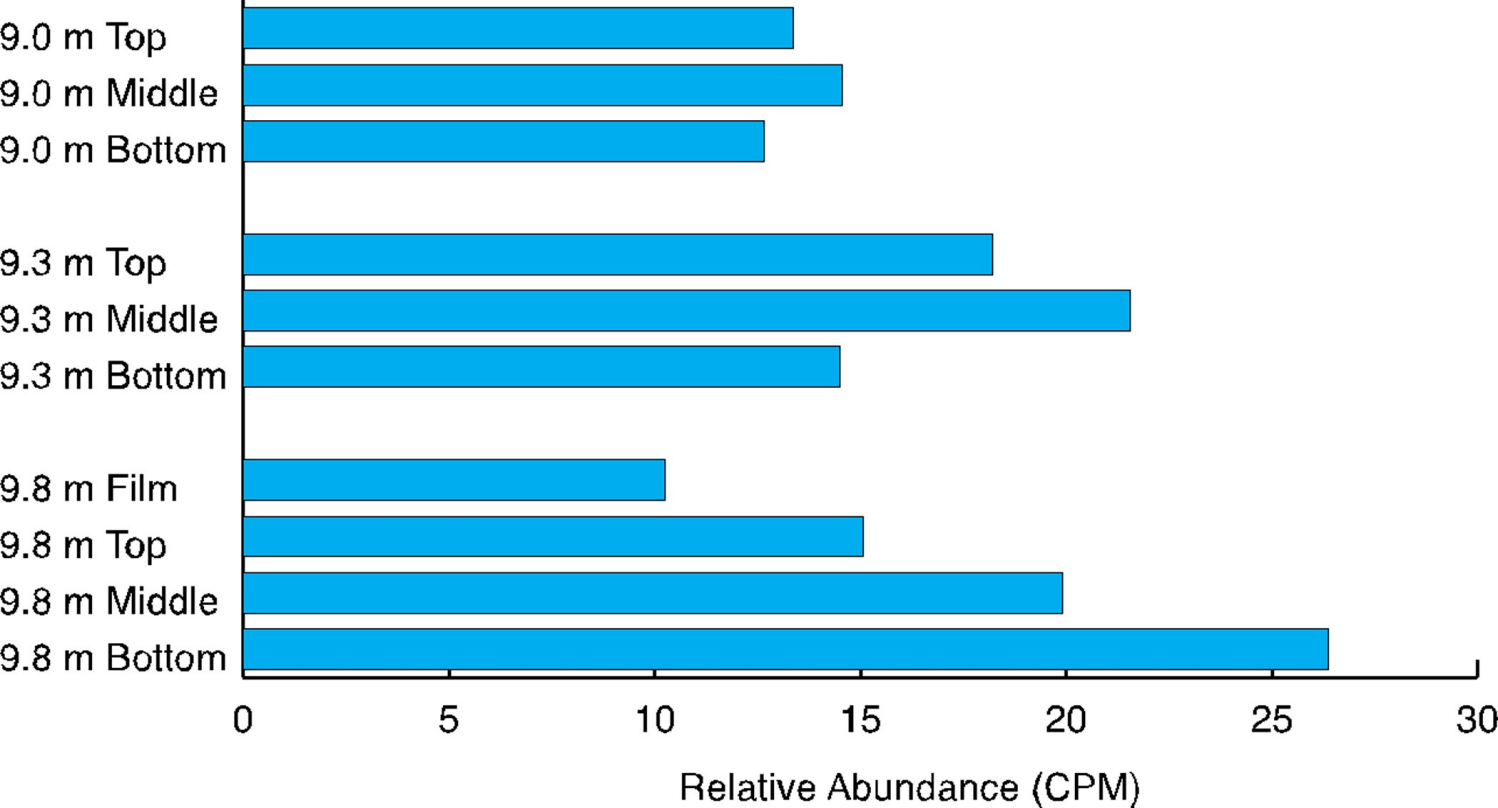
Relative abundance of aerobic respiration capacity. *ccoNO* as estimated from metagenomic sequence data remains consistently high throughout depths and layers.

**Fig 5:**
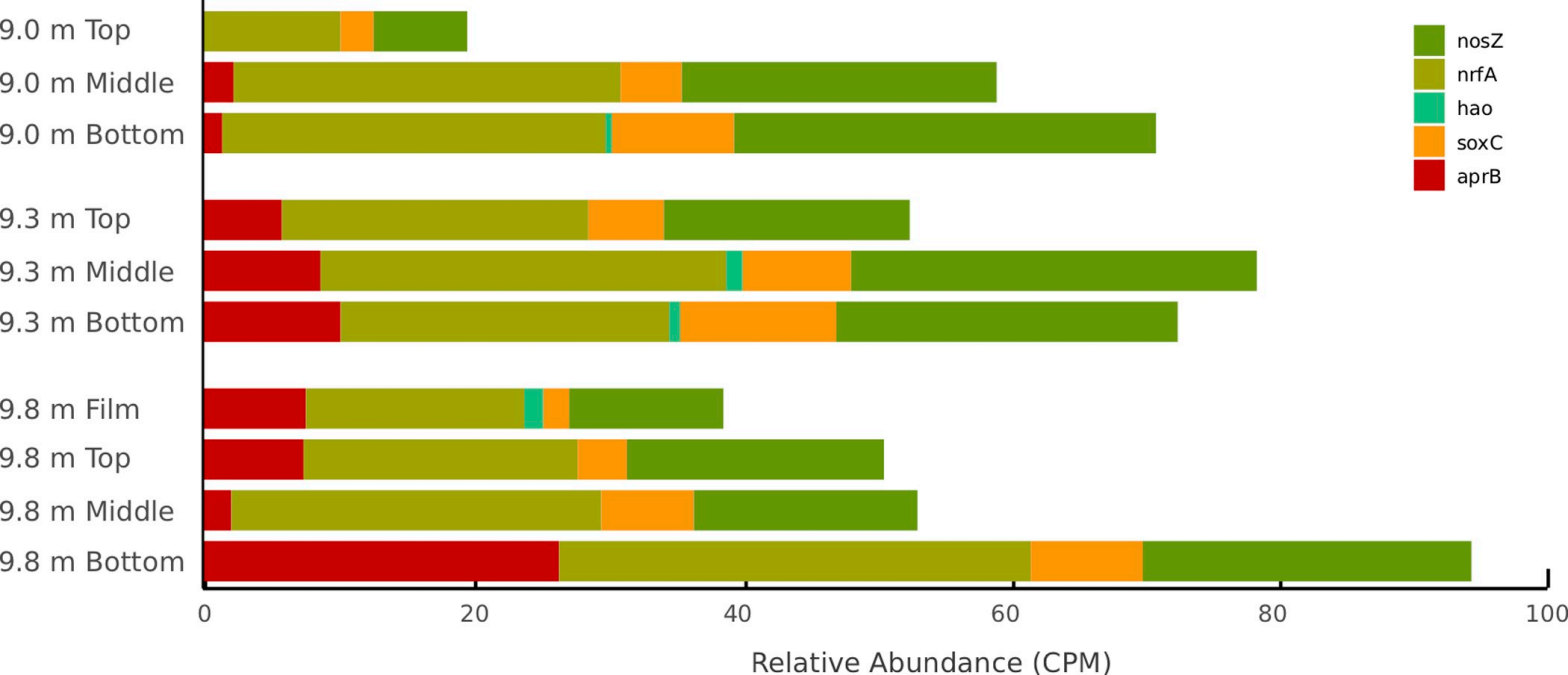
Relative abundance of anaerobic respiration capacity. Anaerobic respiration genes as estimated from metagenomic sequence data. Capacity for nitrogen and sulfur respiration (*nrfA*, *aprB*) increases with depth through mat layers.

With the exception of aerobic respiration (*ccoNO*), the relative abundances of genes encoding respiration and major nutrients such as nitrogen, phosphorus, and sulfur assimilation functions correlated with [O_2_] (Table 5). The capacity for aerobic respiration was consistently high at all depths and in all mat layers, irrespective of environmental availability of O_2_ (Fig 4 and Table 4). Anaerobic respiration genes increased in relative abundance through layers at all depths (Fig 5 and Table 4). Dissimilatory nitrate reduction (*nrfA*) and denitrification (*nosZ*) genes were the most abundant genes encoding the use of electron acceptors other than oxygen (Fig 5 and Table 4). The capacity for sulfate respiration (as indicated by the relative abundance of *soxC*) increased through mat layers at all lake depths (Fig 5). The relative abundance of sulfate reduction via *aprB* was more variable, but also increased through mat layers (Table 4); *aprB* was found in far greater relative abundance in the bottom layer at 9.8 m than in any other sample type (Fig 5 and Table 4).

**Fig 6:**
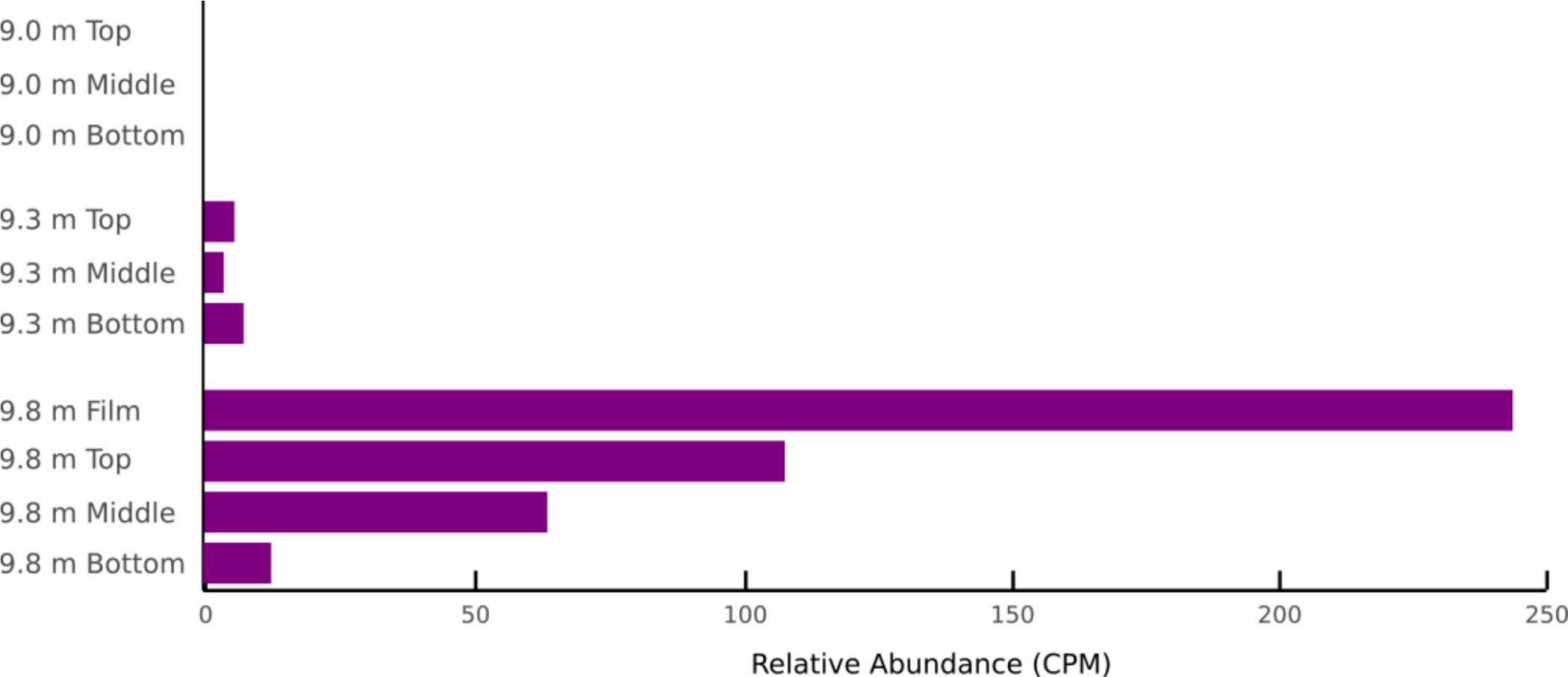
Relative abundance of *hdrB*. Estimated from metagenomic sequence data varies significantly with water depth and among layers at 9.8 m.

The gene *hdrB* is generally associated with methanogenesis but possibly also indicates a capacity for flavin-based electron bifurcation [56]. It was relatively abundant at 9.8 m and also detectable in 9.3 m samples (Fig 6 and Table 4) even though methanogens were not identified in these samples [23]. Tukey’s post-hoc test revealed that *hdrB* relative abundance varied significantly between the 9.8 m film and all layers from 9.0 m and 9.3 m (Table 6). The relative abundance of *hdrB* strongly co-varied with genes for oxygenic photosynthesis and carbon fixation: Pearson’s correlation coefficients between *hdrB* and *psbA* or *rbcL* are 0.897 and 0.877, respectively. The capacity for methanogenesis (*hdrD*) was absent from all samples. Methanotrophy genes (*mdh2*) were only detected in the film at 9.8 m (Table 3).

**Table 6:** Significant differences in metabolic marker genes.

	9.0 m			9.3 m			9.8 m			
	Top	Middle	Bottom	Top	Middle	Bottom	Film	Top	Middle	Bottom
9.0 m										
Top	x									
Middle		x								
Bottom			x							
9.3 m										
Top				x						
Middle					x					
Bottom						x				
9.8 m										
Film	*hdrB* *psbA* *rbcL*	*hdrB* *psbA* *rbcL*	*hdrB* *psbA* *rbcL*	*hdrB* *psbA* *rbcL*	*hdrB* *psbA* *rbcL*	*hdrB* *psbA* *rbcL*	x			
Top	*hdrB* *psbA* *rbcL*	*hdrB* *psbA* *rbcL*	*psbA* *rbcL*	*psbA* *rbcL*	*psbA* *rbcL*	*psbA* *rbcL*	*sbA*	x		
Middle	*psbA*	*psbA*	*psbA*	*psbA*	*psbA*	*psbA*	*hdrB* *psbA*	*psbA*	x	
Bottom							*hdrB* *psbA* *rbcL*	*psbA* *rbcL*	*psbA*	x

Metabolic marker genes from sample metagenomes that vary significantly (adjusted P-values, Tukey’s honest significant difference test) between samples. Entries with P-values greater than 0.01 are not shown and self-comparisons are marked “x”.

**Fig 7:**
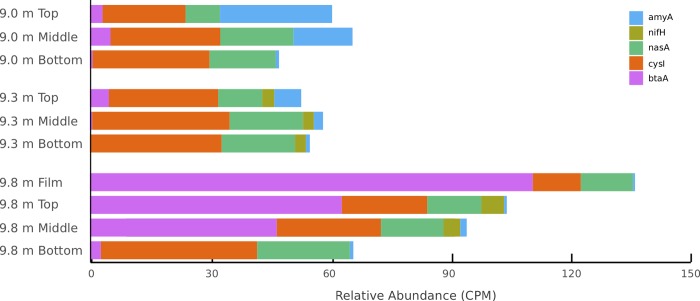
Nutrient assimilation capacity. Predicted capacity differs among lake depth and mat layer, as estimated from metagenomic sequence data. Nitrogen fixation genes (*nifH*) are absent from habitats supersaturated with oxygen as well as the film at 9.8 m, which is seasonally oxic.

Nutrient assimilation trends were specific to lake depth and mat layer. Nitrogen fixation capacity (*nifH*) was absent in 9.0 m samples but present in some 9.3 m and 9.8 m mat layers (Table 4 and Fig 7). Assimilatory nitrate (*nasA*) and sulfate reduction (*cysI*) genes were found in consistent relative abundance throughout all mat layers (Fig 7 and Table 4). Higher relative abundance of the capacity to substitute nitrogenous groups into membrane lipids (*btaA*; [57]), were found in the film, top, and middle layers at 9.8 m (Fig 7 and Table 4).

## Discussion

Photosynthetically active radiation correlated with key metabolic genes in Lake Fryxell, specifically the capacity for oxygenic photosynthesis and carbon fixation. Oxygenic photosynthesis genes are most abundant in the top layers at each depth, consistent with greater PAR at mat surfaces and prior studies of phylogenetic data [22,23]. Photosynthesis requires PAR, so the decreasing relative abundances of *psbA* with layers into the mat and from 9.0 to 9.3 m is consistent with the utility of photosynthesis where there is light (Figs 1 and 3). However, the proportion of *psbA* in surface mat layers did not correlate directly with PAR across all lake depths. The amount of PAR reaching the mats growing at 9.8 m is just above the threshold for net photosynthetic production [22,34], yet samples from the film and top mat layers have the highest relative abundance of *psbA* of all depths (Fig 3). The single Cyanobacterial lineage *Phormidium pseudopristleyi* dominates these samples [22,23]. The high population density of this organism likely explains the disproportionate representation of *psbA*.

### Energy capture and use: Photosynthesis, respiration, and flavin-based electron bifurcation

The high relative abundance of the capacity for oxygenic photosynthesis overall supports previous studies indicating that oxygenic photosynthesis is the most ecologically important energy capture mechanism available to the communities in Lake Fryxell at depths where PAR is available [22,23]. The relative abundances of *psbA* and *rbcL* genes have a Pearson’s correlation coefficient of 0.998 (Table 5), which is consistent with them being hosted in the same organisms, likely Cyanobacteria which are fixing the most carbon and generating the most biomass in the lake. The relative abundance of the capacity for polysaccharide hydrolysis (*amyA*) correlated with those for oxygenic photosynthesis and carbon fixation at only 9.0 m (S4 Table), where mats are oxygenated to a greater extent than at any other depth, and likely throughout the year [34]. Psychrophilic organisms that encode *amyA* are generally aerobes [58–60] and may be more efficient at polysaccharide hydrolysis in oxic environments [61].

The relative scarcity of genes encoding anoxygenic photosynthesis (absence of *pscA* and very low relative abundance of *pufL*) is interesting in the context of previous work indicating that anoxygenic phototrophs are often abundant in low-light environments (*e*.*g*., [62,63]). Anoxygenic phototrophs that use *pufL* are part of the planktonic community in Lake Fryxell [64,65], and also have been detected in MDV Lake Vanda [66], but appear to be absent from MDV Lake Joyce [67]. The low relative abundances of *pufL* and absence of *pscA* may be related to the spectrum of light reaching the benthic surface of Lake Fryxell. The absorption spectrum of bacteriochlorophyll is near 700 nm [68] and the majority of light reaching the mats in ice-covered lakes is shorter wavelength due to increasing attenuation of longer wavelengths with depth [69,70]. The paucity of light at wavelengths suitable for anoxygenic phototrophs may render anoxygenic phototrophy an ineffective metabolic strategy, consistent with both the paucity of *pufL* and *pscA* genes in general, as well as their absence at 9.8 m. In Lake Joyce, the penetration of irradiance through the ice cover is also low, between approximately 0.4% and 4% [67]. In Vanda, approximately 16% of incident irradiance penetrates the ice cove[71]. Thus, it appears that PAR wavelength attenuation contributes to habitat suitability for anoxygenic phototrophs in MDV lakes.

Where sufficient O_2_ is available, aerobic respiration is the most efficient means of ATP generation for organisms. In Fryxell’s benthic mats, no statistically significant difference in the capacity for aerobic respiration, as measured by *ccoNO* relative abundance, exists between habitats where oxygen is constantly available, those where it is seasonally available, and those where it is constantly absent (Fig 4). The widespread capacity for aerobic respiration across [O_2_] in Fryxell mats may be attributable to the fact that bacteria can perform aerobic respiration at nanomolar concentrations of O_2_ using terminal oxidases with a high-affinity for O_2_ (*ccoNO*) [72]. Although the heterogeneity of anoxic environments has not been directly characterized in Fryxell mats, it is likely that micro-oxic and anoxic sub-habitats are more common as oxygen declines with depth in the lake and into the mats [34]. In such habitats, genes for both aerobic and anaerobic respiration are likely maintained because enough oxygen heterogeneity exists both spatially and temporally to make both strategies valuable. Anaerobic respiration using nitrate and sulfate appear to be viable strategies at all depths (Fig 5). The greater relative abundance of nitrogen respiration genes over assimilatory nitrate reduction genes in Fryxell (Table 4) may indicate the importance of nitrogen species as electron acceptors. Testing expression patterns of nitrogen cycling genes in shoulder and winter seasons would allow a better understanding of the effects of strong seasonality, especially availability of PAR and [O_2_], has on these communities.

While photosynthesis and aerobic respiration are the dominant energy metabolisms in Lake Fryxell, mats at 9.8 m show an interesting possible alternative metabolic strategy, as represented by the relative abundance of *hdrB* genes. *hdrB* encodes a subunit of a cytoplasmic complex that reduces two thiol coenzymes [73], which is crucial to methane production in methanogens that have been found in Fryxell’s planktonic community [74,75]. *hdrB* is strictly inhibited by oxygen [76]. However, in Fryxell mats, *hdrB* homologs were found in statistically higher relative abundance in the 9.8 m film sample type (Table 6) where the mats are anoxic only during the winter months [34]. Phylogenetic markers of methanogens are absent in samples with high relative abundances of *hdrB* [22,23], suggesting *hdrB* is hosted in non-methanogens. Interestingly, *hrdB* is present in some sulfate reducing bacteria [77–79] and may be necessary for energy generation among diverse anaerobes [56]. In these organisms, *hdrB* is part of an enzyme complex called flavin-based electron bifurcation that acts as an alternative to both substrate level phosphorylation (fermentation) and electron transport [80]. In Fryxell mats, *hdrB* appears to mark capacity for flavin-based electron bifurcation in sulfate reducers rather than methane production, the first ecological evidence of this function of *hdrB* to our knowledge.

### Nutrient cycling and limitation

Nitrogen fixation capacity in Lake Fryxell appears to be limited by local [O_2_] as *nifH* is absent from mats continuously exposed to oxic water (Fig 4). Typically in microbial mats, nitrogen fixation and ammonium and nitrate assimilation are performed by community members living near the surface of a mat that is illuminated and oxygenated [81], particularly by *Nostoc* spp. [82]. Many Antarctic mat ecosystems have a greater apparent capacity for nitrogen fixation than we found here, especially where *Nostoc spp* are in high abundance [81]. However, *Nostoc spp*. are rare in Fryxell’s mats [22,23]. Nitrogen fixation in non-heterocystous cyanobacteria occurs at night, when oxygen is no longer being generated and depleted from the cells [83,84]. The absence of dark conditions during the Antarctic summer leads to the continuous production of oxygen by cyanobacteria, which inhibits nitrogen fixation. Thus the polar latitude of Lake Fryxell may significantly limit nitrogen fixation above the oxycline even if the communities contained the capability to do so, consistent with previous metagenomic results [85]. Further, Fryxell's water column above the oxycline contains less than 1 μg / L nitrate or ammonium [22], leading to the hypothesis that the planktonic microbial community is also limited by nitrogen [20]. Given the low relative abundance of *nifH*, Lake Fryxell mats above the oxycline are also likely nitrogen limited, whereas water column nitrate and ammonium levels rise below the oxycline [22]. In contrast to the likely inhibition of nitrogen fixation in the O_2_ supersaturated mats at 9.0 m, the absence of *nifH* in the top layer at 9.8, where mats are only weakly oxic seasonally, may be due to the high population density of the *Phormidium*, which often lacks the ability to fix nitrogen [86]. In the bottom layer at 9.8 m, where the capacity for nitrogen fixation could be attributable to heterotrophic bacteria [87], the absence of *nifH* is likely due to low availability of energy for nitrogen fixation, which requires an abundance of ATP [88]. In contrast, the low-light environment in the bottom layers at 9.3 m may provide enough PAR to support nitrogen fixation, and *nifH* is detectable in this layer (Fig 7).

Nitrogen and phosphorus cycling in planktonic communities in Lake Fryxell were recently investigated by [89], who found evidence that nitrogen and phosphorus are co-limiting. The relative availability of nitrogen versus phosphorus can affect the substitution of nitrogenous groups for phosphate groups in membrane lipids [57], a process that requires the gene *btaA*. The increased relative abundance of membrane phosphorus substitution genes at 9.8 m relative to samples with lower predicted nitrogen availability may indicate a switch in nutrient limitation from nitrogen to phosphorus at the oxycline. Mats growing below the oxycline in Fryxell have nitrogen available to them both through nitrogen fixation via *nifH* and water column nitrate and ammonium levels rise faster than dissolved reactive phosphorus below the oxycline [22]. Thus, variations in water column chemistry and the distribution of *btaA* indicate that there is likely spatial variability in nutrient availability.

In contrast to nitrogen cycling, microbial sulfur cycling occurs across a range of oxygen concentrations, and sulfur oxidation and reduction are typically performed throughout microbial mats [83]. Assimilatory sulfate reduction is required for incorporation of sulfur into amino acids (biomass) in the absence of sulfide, whereas dissimilatory sulfate reduction is a means of anaerobic respiration. In general, dissimilatory sulfate reduction is an important anaerobic metabolism in microbial mats, especially where cyanobacteria generate low molecular-weight organics as substrates [84]. However, assimilatory sulfate reduction genes are found in greater relative abundance than dissimilatory sulfate reduction genes in Lake Fryxell (Figs 5 and 7 and Table 4). The difference in relative abundance of sulfate reduction genes in Fryxell mats may indicate that sulfate is primarily used for biomass generation rather than respiration.

### The species sorting model applied to metabolic composition and diversity

Analyses of taxonomic composition and phylogenetic diversity suggested that the species sorting model is the most appropriate for describing benthic mat structure in Lake Fryxell across large- and small-scale PAR and [O_2_] gradients [23]. Therefore, we expected the metabolic strategies of the mat communities to also closely match the local heterogeneity of PAR and [O_2_] at the millimeter- and meter-scales. Understanding the metabolic capacity of the Fryxell's mat communities across the gradients of PAR input and [O_2_] is crucial to understanding the processes driving community composition because fitness is dictated by individuals' traits.

Gene family diversity trends support the hypothesis that the species sorting model can be appropriately applied to the communities in Lake Fryxell. We found that gene family diversity increased at the meter-scale across the lake floor and at the millimeter-scale through mat layers at 9.0 and 9.3 m, negatively correlating with PAR. Likely, the genes needed for oxygenic phototrophy, the dominant metabolic strategy in the top layers at 9.0 and 9.3 m (Table 4 and Fig 3), suppress gene family diversity, which is relieved as phototrophy becomes less dominant through mat layers. This is consistent with phylogenetic and taxonomic results of these samples [22,23], and supports the interpretation that the communities through the layers at 9.0 and 9.3 m are organized to maximize energy capture [23,24]. The proportions of metabolic genes change as PAR decreases, indicating that the metabolic capacity of the mats at 9.0 and 9.3 m is structured by the local environmental conditions. In contrast, gene family diversity decreased through mat layers at 9.8 m, where [O_2_] varies the most seasonally. Gene family diversity is also greatest in the film at 9.8 m. Samples from the top layer at 9.8 m show strong negative correlation between phylogenetic diversity and gene family diversity (Pearson correlation coefficient -0.790). The phylogenetic diversity in this habitat is quite low, likely due to the highly selective environmental conditions [23]. This implies that in this seasonally illuminated, seasonally oxic, low-energy, sulfidic environment, gene family diversity is important for survival as habitat conditions change throughout the year. Future investigation into how gene family diversity is distributed among community members in the film and top layers at 9.8 m will likely provide further insight into tradeoffs between fitness and diversity in this habitat.

The metabolic marker genes that varied significantly between different local [O_2_] and PAR input are those most important for optimization of energy capture. The relative abundances of genes encoding oxygenic photosynthesis (*psbA*) and carbon fixation (*rbcL*) at 9.8 m are greatest where high populations of Cyanobacteria capture the energy available at the mat surface. Cyanobacteria produce O_2_, which drives aerobic respiration and supports other, lower energy metabolisms when the mats become anoxic over winter. For example, organic carbon fixed by photoautotrophs likely supplies the substrates required by organisms using flavin-based electron bifurcation (*hrdB*), which is O_2_-inhibited and would be active only in the winter. The potential metabolic strategies of Fryxell mats across environments with different energy inputs suggest that they have maximized energy capture consistent with the maximum power principle [24,90] and the *species sorting* model.

Alternative models within the metacommunity framework do not explain the patterns of metabolic diversity and composition in Fyrxell’s benthic mats. The *patch dynamics* model is inappropriate to Lake Fryxell because it requires local habitats conditions to be uniform, which does not conform to variability in PAR and [O_2_] with depth in Lake Fryxell. The *mass effects* model would suggest that the metabolic composition of communities on the surface of the mats at each depth would be similar to that of the nearby lake water due to the settling of microorganisms. However, the benthic community is strikingly different from the planktonic community; specifically, the planktonic community contains abundant and diverse purple phototrophic bacteria [65], which are absent from the benthic microbial mats. The *neutral model* would be expected to produce communities that might vary in their metabolic diversity but without any relationship to environmental conditions, and therefore fails to explain the patterns of marker gene distribution along the PAR and [O_2_] gradients in Lake Fryxell.

Self-organizing systems such as these microbial communities are structured by their environment across both spatial and temporal scales; the relative abundances of species housing specific metabolic strategies adjust in population to achieve maximum power input given average energy availability throughout the year, with depth into the lake and through mat layers. Phototrophic and heterotrophic populations in Lake Fryxell’s benthic community likely change differently over the course of the annual PAR cycle because they occupy different niches. Phototrophs require PAR, and so likely increase in activity in the spring and summer. In the winter, phototrophs generally respond by a combination of entering dormant states, enduring reduced population abundances and loss of biomass via cell death, and shifting to heterotrophy or fermentation [91]; in MDV lakes, phototrophs may also be buried in mat over years rather than seasons [67,92]. Heterotrophs, and mixotrophs (seasonally), rely on organic carbon reservoirs built up over the years by the autotrophs. Heterotroph and mixotroph populations in the benthic mats likely shift according to organic carbon quality and quantity throughout the summer and winter, as do populations in the pelagic community [33,93]. Additionally, both phototrophic and heterotrophic populations living at 9.0 m likely change differently than those at 9.8 m. At 9.0 m, the O_2_ saturation of the mats makes aerobic respiration available year-round. But at 9.8 m, the mats are predicted to become anoxic during winter, so other electron acceptors then become important. The increased relative abundance of extremely low-energy strategies such as flavin-based electron bifurcation via *hdrB* at 9.8 m (Fig 6) are evidence that annual variation in PAR further affects the metabolic strategies found in the mats according to local environmental heterogeneity, in this case seasonal energy availability. The metabolic patterns uncovered here are consistent with the species sorting model because spatial and temporal heterogeneity of physicochemical characteristics (PAR, [O_2_], nitrate, phosphorus, *etc*.) explain patterns of metabolic genes in Fryxell’s benthic mats. Independent evidence suggests that OTU abundances optimize energy capture in Fryxell’s planktonic community [75], and the same is true for Fryxell’s benthic community.

An even more extreme example of the applicability of the species sorting model to microbial communities may be found in hot springs in Yellowstone National Park. The hot springs are considerably more constrained than Lake Fryxell, both phylogenetically and metabolically, where the dominant phylogenetic lineage may compose between 63 and 100% by SSU amplicon analyses and [O_2_] limitation favors hydrogen metabolisms [94,95]. In contrast, the microbial mats growing in Guerrero Negro are phylogenetically stratified, likely according to PAR and geochemical gradients [96,97]. At Guerrero Negro, the chemical complexity of the habitat allowed the phylogenetic diversity to map onto environmental heterogeneity. The Guerrero Negro mats are therefore more similar to the stratified and stably heterogeneous environment of Lake Fryxell. These habitats differ in environmental conditions, but all demonstrate the applicability of the species sorting model, and metacommunity theory generally, to frame future research in extreme environments and microbial mat ecosystems.

## Conclusions

Assessment of the gene family diversity and metabolic marker genes indicates that PAR and [O_2_] control the distribution of potential metabolic strategies in Lake Fryxell. A multivariate statistical analysis of the relative abundance of metabolic marker genes shows that oxygenic photosynthesis, carbon fixation, and flavin-based electron bifurcation are the key metabolic strategies that differentiate mats growing in different environmental sub-habitats. Metabolic marker genes for anaerobic respiration likely result from spatial and temporal heterogeneity in [O_2_] in Lake Fryxell. Further, the high relative abundance of *btaA* suggests that microbial mats in Fryxell appear to be phosphorus-, not nitrogen-limited in the anoxic portion of the lake, consistent with water column concentrations of nitrite, nitrate, and soluble reactive phosphorus. Attenuation of red light with depth may explain the dearth of anoxygenic photosynthesis genes. Finally, the pattern of gene family diversity through the mat layers and metabolic marker gene relative abundances of *psbA*, *rbcL*, and *hdrB* correlate strongly with PAR and [O_2_] and point to the importance of their seasonal fluctuation.

The spatial heterogeneity of PAR and [O_2_] in Lake Fryxell provide the foundation for the organisms in Lake Fryxell to organize according to metabolic diversity and composition, similar to their phylogenetic structure [23], supporting the maximum power principle as applicable in this microbial ecosystem. More broadly, the species sorting model appears to be applicable to the metacommunity in Lake Fryxell as regards both phylogenetic lineages [23] and metabolic traits because niche selection (via the maximum power principle) governs which lineages and metabolic marker genes are found in which habitats.

## Supporting information

S1 Table Samples collected. Summary of samples collected in November 2012 from benthic microbial mats in Lake Fryxell, Antarctica.
(XLSX)

S2 Table Sequencing statistics of samples. (XLSX)

S3 Table Correlation between phylogenetic and metabolic diversity. Pearson's correlation coefficient between phylogenetic diversity (bwpd) [23] and metabolic diversity (Simpson's Index of Diversity calculated from gene family abundances as called by Humann2). Both measures of alpha diversity were estimated from metagenomic sequences.
(XLSX)

S4 Table Correlations among amyA, psbA, and rbcL at 9.0 m. (XLSX)

S5 Table CPM table. CPM by replicate.
(XLSX)
